# Risk factors for carbapenem-resistant *Klebsiella pneumoniae* infection relative to two types of control patients: a systematic review and meta-analysis

**DOI:** 10.1186/s13756-020-0686-0

**Published:** 2020-01-31

**Authors:** Wei-min Zhu, Zhe Yuan, Hong-yu Zhou

**Affiliations:** 1 Division of Infectious Diseases, The First Affiliated Hospital of Chongqing Medical University, No. 1 You Yi Road, Yuan Jia Gang, Yuzhong District, Chongqing, 400016 China; 2 Department of Hospital Infection Control, The First Affiliated Hospital of Chongqing Medical University, No. 1 You Yi Road, Yuan Jia Gang, Yuzhong District, Chongqing, 400016 China

**Keywords:** *Klebsiella pneumoniae*, Carbapenem-resistance, Infection, Risk factor, Systematic review, Meta-analysis

## Abstract

**Background:**

Studies on risk factors for carbapenem-resistant *Klebsiella pneumoniae* (CRKP) infection have provided inconsistent results, partly due to the choice of the control group. We conducted a systematic review and meta-analysis to assess the risk factors for CRKP infection by comparing CRKP-infected patients with two types of controls: patients infected with carbapenem-susceptible *Klebsiella pneumoniae* (comparison 1) or patients not infected with CRKP (comparison 2).

**Methods:**

Data on potentially relevant risk factors for CRKP infection were extracted from studies indexed in PubMed, EMBASE, Web of Science or EBSCO databases from January 1996 to April 2019, and meta-analyzed based on the outcomes for each type of comparison.

**Results:**

The meta-analysis included 18 studies for comparison 1 and 14 studies for comparison 2. The following eight risk factors were common to both comparisons: admission to intensive care unit (ICU; odds ratio, OR_comparison 1_ = 3.20, OR_comparison 2_ = 4.44), central venous catheter use (2.62, 3.85), mechanical ventilation (2.70, 4.78), tracheostomy (2.11, 8.48), urinary catheter use (1.99, 0.27), prior use of antibiotic (6.07, 1.61), exposure to carbapenems (4.16, 3.84) and exposure to aminoglycosides (1.85, 1.80). Another 10 risk factors were unique to comparison 1: longer length of hospital stay (OR = 15.28); prior hospitalization (within the previous 6 months) (OR = 1.91); renal dysfunction (OR = 2.17); neurological disorders (OR = 1.52); nasogastric tube use (OR = 2.62); dialysis (OR = 3.56); and exposure to quinolones (OR = 2.11), fluoroquinolones (OR = 2.03), glycopeptides (OR = 3.70) and vancomycin (OR = 2.82).

**Conclusions:**

Eighteen factors may increase the risk of carbapenem resistance in *K. pneumoniae* infection; eight factors may be associated with both *K. pneumoniae* infections in general and CRKP in particular. The eight shared factors are likely to be ‘true’ risk factors for CRKP infection. Evaluation of risk factors in different situations may be helpful for empirical treatment and prevention of CRKP infections.

## Background

Carbapenem-resistant Gram-negative bacteria, mainly *Klebsiella pneumoniae*, are an emerging cause of healthcare-associated infections that pose a significant threat to public health [1]. The percentage of *K. pneumoniae* infections resistant to carbapenems continues to rise [2, 3], with proportions exceeding 50% in parts of the Eastern Mediterranean and Europe [1, 2]. *K. pneumoniae* carbapenemase originated in the northeastern USA in the early 2000s, but rapidly disseminated to other regions worldwide [4].

Carbapenem-resistant *K. pneumoniae* (CRKP) infection is difficult to treat since carbapenems are often considered last-resort antibiotics for severe *K. pneumoniae* infections. The most important genes that can confer carbapenem resistance (via carbapenemases) are present in *K. pneumoniae*, rendering almost all available treatment options ineffective [2]. Mortality rates reach 33–50% among CRKP-infected patients in different regions of the world [5], significantly higher than mortality caused by infection with carbapenem-susceptible *K. pneumoniae* (CSKP) [1]. Preventing CRKP infection is therefore important not only to avoid poor prognosis and even death, but also to prevent widespread transmission of carbapenem resistance through mobile genetic elements [6, 7].

Numerous studies have assessed risk factors for CRKP infection with different and sometimes even contradictory conclusions. A previous meta-analysis attempted to address this inconsistency [8] but did not take into consideration that different studies often use different control (reference) groups. The appropriate selection of the control group in the analysis of risk factors for antibiotic-resistant pathogen infections depends on the specific research question [9–12]. In studies analyzing risk factors for CRKP infection, two control groups are most often selected: patients infected with CSKP or patients without CRKP infection. The comparison of CRKP-infected with CSKP-infected patients may allow the identification of risk factors for carbapenem-resistant infections, although the results may be overestimated. In contrast, the comparison of CRKP-infected individuals with patients without CRKP infection may help to identify risk factors associated with both *K. pneumoniae* infections in general and CRKP in particular. Risk factors that are significant in both comparisons can be considered ‘true’ risk factors for CRKP infection [11, 12].

Thus, we performed a systematic review and meta-analysis to clarify risk factors for CRKP infection relative to infection with CSKP (comparison 1) or to the absence of CRKP infection (comparison 2). This design, similar to a case-control-control study, aimed to compare the results of the two analyses and their different implications for the clinical practice, allowing the identification of the likely true risk factors for CRKP infection.

## Methods

This meta-analysis was conducted according to the Preferred Reporting Items for Systematic Reviews and Meta-Analyses (PRISMA) guidelines [13].

### Search strategy

Two authors (H.Y.Z. and Z.Y.) searched for relevant studies in PubMed, EMBASE, Web of Science and EBSCO databases that were published from January 1996 to April 2019. The search terms included “*Klebsiella pneumoniae*” AND (“carbapenem-resistant” OR “imipenem-resistant” OR “meropenem-resistant” OR “ertapenem-resistant” OR “carbapenemase-producing” OR “*Klebsiella pneumoniae* carbapenemase”) AND (“risk factors” OR “risk” OR “factors”). Only studies published in English were considered. Reference lists in selected articles and relevant review articles were manually searched to identify additional studies.

### Inclusion and exclusion criteria

Studies were included if they met the following criteria: (1) case-control or cohort study design, whether prospective or retrospective; (2) the risk factors for CRKP infection were reported; (3) either comparison 1 or comparison 2 was made; (4) CRKP and CSKP were classified based on *K. pneumoniae* isolate identification and tests for resistance to carbapenem (imipenem, meropenem, or ertapenem) involving well-defined microbiological methods; and (5) infection was explicitly defined. The inclusion criterion (3) led us to exclude studies comparing patients infected with carbapenemase-producing *K. pneumoniae* (CPKP) with controls without such infection, since such controls may have been infected with carbapenem-resistant, non-carbapenemase-producing *K. pneumoniae*. Studies were also excluded if they had the format of a report, review, comment, meeting abstract or letter to the editor; or if they reported insufficient data to assess outcomes.

### Data extraction

Two authors (H.Y.Z. and W.M.Z.) independently evaluated and extracted data from the included studies using a predefined, standardized protocol. The extracted data on general characteristics of studies included the first author’s name, year of publication, journal of publication, country, study period, study design and setting, type of inter-group comparison, sample size, average age, and sex distribution. Potential risk factors were included in the meta-analysis only if at least three studies examined them and those studies reported the numbers of individuals in each comparison group. Disagreements about extracted data were resolved through discussion.

### Quality assessment

Two authors (W.M.Z. and Z.Y.) independently evaluated the quality of each study using the Newcastle-Ottawa Scale (NOS), a scale for assessing the quality of published non-randomized studies in meta-analyses [14]. The scale contains eight items, categorized into three dimensions: selection, comparability, and outcome (cohort studies) or exposure (case-control studies) [14]. We developed a NOS-based scale ranging from 0 to 9 points: studies scoring 0–4 points were defined as low quality, while those scoring 5–9 points were defined as high quality. Differences were resolved by consensus.

### Statistical analysis

The meta-analysis was performed using RevMan 5.2 software provided by The Cochrane Collaboration (Copenhagen: The Nordic Cochrane Centre, 2014). Pooled odds ratios (ORs) and 95% confidence intervals (CIs) were calculated for all outcomes. The *Z*-test was used to determine the significance of the pooled OR, and the results were considered statistically significant when *P* < 0.05. Statistical heterogeneity among studies was assessed using a chi-squared test in which *P* < 0.10 was taken as the threshold for significant heterogeneity, or by calculating *I*^*2*^ value, with *I*^*2*^ > 50% considered evidence of heterogeneity [15]. Depending on the assessed heterogeneity, the Mantel-Haenszel fixed- or random-effect methods were used to meta-analyze the outcomes.

Publication bias was quantitatively analyzed using Egger’s test in STATA software version 12.0 (College Station, TX: StataCorp LP) [16], and the results were considered statistically significant when *P* < 0.05. Sensitivity analyses were conducted by omitting studies one by one, and the *P* values of pooled ORs were compared. The results were considered robust when the *P* values were not substantially different.

## Results

### Study selection

**Fig. 1:**
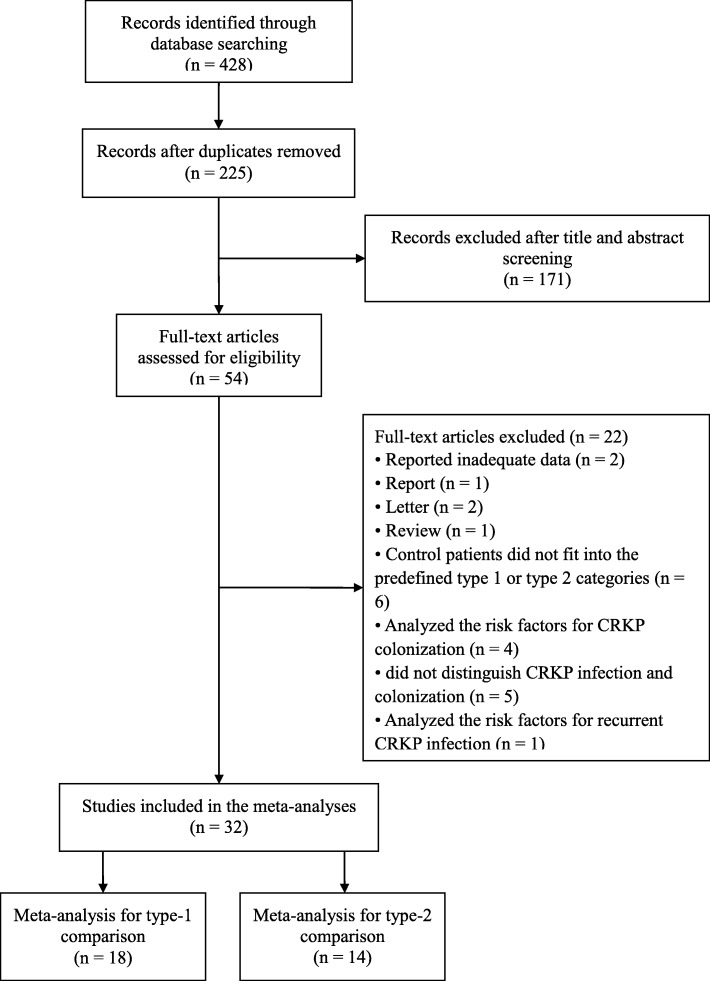
Flow diagram of study selection for meta-analysis. Abbreviations: CRKP, carbapenem-resistant *Klebsiella pneumoniae*; CSKP, carbapenem-susceptible *Klebsiella pneumoniae*

A total of 428 unique records were retrieved from electronic databases, and 203 duplicate records were removed. After screening of titles and abstracts, 171 records were excluded. The remaining 54 studies were read in full to determine the eligibility. In the end, 18 studies performing comparison 1 [17–34] and 14 for comparison 2 [35–48] were included in the systematic review, while subsets of these studies were included in the meta-analyses of the various risk factors (Fig. 1).

### Study characteristics

The main characteristics of the 18 studies included in comparison 1 are presented in Table 1. The studies were published from 2007 to 2019, and involved 1010 patients with CRKP infection and 1190 with CSKP infection from nine countries: China (6 studies), Greece (3), Israel (2), USA (2), Italy (1), Colombia (1), Turkey (1), Brazil (1), and Georgia (1). The designs of the 18 studies were case-control (12), retrospective cohort (3), case-case-control (1), nested case-control (1), and prospective cohort (1). The comparison and reference groups were matched in 11 studies. All but three studies enrolled patients from a single center, and six studies enrolled only patients in the intensive care unit (ICU).

**Table 1:** Characteristics of studies included in the meta-analysis of the type 1 comparison

Study	Study design	Matching ratio	Matched factors	Enrollment period	Country	Setting	Sample size, CRKP infection/CSKP infection	Average age(SD or range),CRKP infection/CSKP infection	Sex (male), CRKP infection/CSKP infection	NOS points
Gómez, 2014 [7]	Case-case-control	1:1:2	Length of stay in ICU and date of bacterial isolation	January 2008–January 2011	Colombia	Single center	61/61	42.2 ± 28.4/40.5 ± 28.2	30/44	8
Wu, 2011 [8]	Case-control	1:2	Site of infection and the date of hospital admission (± within 5 days)	July 2006–July 2008	China	Single center	39/78	64.0 ± 16.0/56.9 ± 17.6	28/60	7
Falagas, 2007 [9]	Case-control	1:1	Site of infection, age ± 5 years and length of hospital stay up to isolation of CRKP ±3 days and year of hospital admission	October 2000–May 2006	Greece	Multicenter (2 hospitals)	53/53	61.5 ± 18.8/61.9 ± 17.2	23/54	6
Patel, 2008 [20]	Case-control	1:1	Anatomic site of infection, age and date of isolation of *K. pneumoniae*	July 2004–June 2006	USA	Single center	99/99	60.67 ± 14.95/59.39 ± 13.34	58/58	7
Simkins, 2014 [21]	Case-control	NA	NA	January 2006–December 2010	USA	Single center	13/39	53 ± 18/55 ± 16	7/14	5
Hu, 2016 [22]	Case-control	1:1	Year of ICU admission and site of infection	January 2011–June 2013	China	Single center, a 67-bed ICU	65/65	64.12 ± 13.69/59.06 ± 14.61	45/50	6
Candevir, 2015 [23]	Retrospective cohort	NA	NA	January 2012–December 2012	Turkey	Single center, ICUs	47/51	38 (0–83)/8 (0–86) ^a^	31/30	7
Vardakas, 2015 [24]	Retrospective cohort	NA	NA	January 2006–October 2009	Greece	Single center, an 8-bed ICU	73/18	66.3 ± 14.4/60.9 ± 15.6	36/7	7
Correa, 2013 [25]	Case-control	1:2	Infection date, anatomic site of infection, and the unit where infection was acquired	January 2006–August 2008	Brazil	Single center	20/40	59.6/64.9 ^b^	13/21	7
X. Zheng, 2017 [26]	Case-control	NA	NA	January 2013–December 2014	China	Single center, 30-bed medical ICU	31/17	57.61 ± 14.78/62.71 ± 16.34	27/11	5
Zheng, 2017 [27]	Case-control	1:1	In the same ward during the same period (within 30 days) and ages within 5 years of each other	January 2013–July 2015	China	Single center	51/51	69.84 ± 18.0/67.25 ± 20.1	39/35	8
Shilo, 2013 [28]	Case-control	1:1	Hospitalized during the same year	January 2006–April 2009	Israel	Single center	135/127	77 ± 14/80 ± 13	62/53	7
Wang, 2018 [29]	Case-control	1:1	Admitted to the same department during the same time period	January 2010–December 2014	China	Single center	48/48	67.7 ± 19.5/63.1 ± 17.8	35/34	6
Mouloudi, 2010 [30]	Nested case-control	NA	NA	January 2007–December 2008	Greece	Single center, 8-bed polyvalent ICU	37/22	NA	28/17	6
Hussein, 2013 [31]	Case-control	NA	NA	January 2006–December 2008	Israel	Single center	103/214	61.4 ± 17/63.2 ± 18	73/133	7
Pan, 2019 [32]	Retrospective cohort	1:2	Age, sex, and specimen source	2014	China	Single center	66/132	58.8 ± 15.9/57.4 ± 14.7	45/90	8
Tsereteli, 2018 [33]	Case-control	NA	NA	January 2017–February 2018	Georgia	Multicenter (2 hospitals), ICUs	20/26	52.3 ± 19.153/54.46 ± 18.591	18/16	6
Hoxha, 2016 [34]	Prospective cohort	1:1	Age (10 years), hospital, and type of specimen (blood/bronchoscopy specimen)	November 2012–July 2013	Italy	Multicenter (10 Italian hospitals)	49/49	72/74 ^c^	32/32	8

*Abbreviations*: *CRKP* carbapenem-resistant *Klebsiella pneumoniae*, *CSKP* Carbapenem-susceptible *Klebsiella pneumoniae*, *SD* Standard deviation, *NOS* Newcastle-Ottawa Scale, *ICU* Intensive care unit, *NA* Not available

^a^Age, median (range), years

^b^Age, mean, years

^c^Age, median, years

The main characteristics of the 14 studies included in comparison 2 are presented in Table 2. These studies were published from 2012 to 2019, and involved 893 patients with CRKP infection and 3073 without CRKP infection from six countries: Italy (6), USA (2), Greece (2), Turkey (2), Israel (1), and China (1). The designs of the studies were case-control (6), retrospective cohort (4), prospective cohort (2), case-case-control (1), and case-cohort (1). In six of these studies the comparison and reference groups were matched. All but one study enrolled patients from a single center and three studies involved only patients in the ICU.

**Table 2:** Characteristics of studies included in the meta-analysis of the type 2 comparison

Study	Study design	Matching ratio	Matched factors	Period	Country	Setting	Sample size, CRKP infection/without CRKP infection	Average age (SD or range), CRKP infection/without CRKP infection	Sex (male), CRKP infection/without CRKP infection	NOS points
Mouloudi, 2014 [37]	Prospective cohort	1:2	During the same period	January 2008–December 2011	Greece	Single center, 8-bed polyvalent ICU	17/34	54 (44–66)/55 (26–66)^a^	10/19	5
Giannella, 2015 [38]	Prospective cohort	NA	NA	June 2010–December 2013	Italy	Single center	20/217	63 ± 2.8/55 ± 14	15/143	7
Akgul, 2016 [39]	Case-control	NA	At least 72 h in the same wards and period with the cases	January 2010–September 2014	Turkey	Single center	95/100	66 (19–94)/58 (21–87)^a^	63/62	6
Giannella, 2014 [36]	Case–control	1:4	The time of the primary positive CRKP rectal swab (within the same month) and the time atrisk of having a subsequent infection	January 2012–December 2013	Italy	Multicenter (5 large tertiary-care teachinghospitals)	143/572	65 (52–75)/70 (58–81)^a^	84/307	6
Borer, 2012 [35]	Case-control	1:2	Age within 5 years, same sex, time of admission ± 5 days, and similar length of time at risk ±2 days	May 2007–January 2010	Israel	Single center	42/84	72 (19–91)/72.5 (21–95)^a^	NA	6
Yang, 2016 [40]	Case-control	1:2	Month of admission, ward, as well as interval days (interval from admission to confirmation of the index culture)	January 2012–December 2013	China	Single center	370/740	85 (80–87)/74 (59–84)^a^	321/434	7
Micozzi, 2017 [41]	Retrospective cohort	NA	NA	24 February 2012–31 May 2013	Italy	Single center	11/8	NA	5/8	5
Mazza, 2017 [42]	Retrospective cohort	NA	NA	January 2012–December 2015	Italy	Single center	8/302	NA	NA	6
Varotti, 2017 [43]	Case-control	1:2	The patient transplanted chronologically before and the patient transplanted chronologically after the study patient	January 2010–June 2015	Italy	Single center	26/52	59 ± 13/53 ± 14	21/43	8
Salsano, 2016 [44]	Retrospective cohort	NA	NA	January 2104–December 2014	Italy	Single center	32/521	74 (67–77)/71 (63–77)^a^	17/362	6
Kontopoulou, 2019 [45]	Case-cohort	NA	NA	June 2011–August 2014	Greece	Single center, 8-bed medical and surgical ICU	48/178	60/63^c^	NA	6
Gallagher, 2014 [46]	Case-case-control	1:1	Location (hospital unit) and time (within 30 days)	June 2005–October 2010	USA	Single center	43/43	56/58^b^	26/26	6
Kalpoe, 2012 [47]	Retrospectivecohort	NA	NA	1 January 2005–1 October 2006	USA	Single center	14/161	57 (52–71)/55 (23–78)^a^	9/133	6
Akturk, 2016 [48]	Case-control	NA	NA	January 2010–December2014	Turkey	Single center, pediatric and neonatal ICUs	24/61	53 ± 14.7/23.5 ± 5.8	NA	6

*Abbreviations*: *CRKP* Carbapenem-resistant *Klebsiella pneumoniae*, *SD* Standard deviation, *NOS* Newcastle-Ottawa Scale, *ICU* Intensive care unit, *NA* Not available

^a^Age, median (range), years

^b^Age, mean, years

^c^Age, median, years

### Quality assessment

All studies in the review were judged to be of high quality based on NOS assessment. The 18 studies in comparison 1 scored an average of 7 (range 5–8) (Table 1). The 14 studies in comparison 2 scored an average of 6 (range 5–8) (Table 2).

#### Risk factors for CRKP infection based on CRKP-CSKP comparison (comparison 1)

Table 3 shows the risk factors for CRKP infection for this comparison, as well as the heterogeneity in the meta-analysis. All 43 risk factors were dichotomous variables except for the following continuous variables: length of hospital stay (LOS), length of ICU stay, and Acute Physiology and Chronic Health Evaluation (APACHE) II score on ICU admission. Of the 43 factors, the following 18 were statistically significant: longer LOS, prior hospitalization (within the previous 6 months), admission to ICU, renal dysfunction, neurological disorders, tracheostomy, mechanical ventilation, central venous catheter (CVC) use, urinary catheter use, nasogastric tube use, implementation of dialysis, prior use of any antibiotic, and specific use of carbapenems, aminoglycosides, quinolones, fluoroquinolones, glycopeptides, or vancomycin.

**Table 3:** Meta-analysis of risk factors for CRKP infection in the type 1 comparison

	Number of included studies	Sample size, CRKP infection/CSKP infection	Heterogeneity			Effects model	OR or MD [95% *CI*]	*Z*	*P*	Egger’s test, *P* >|t|
			χ^*2*^	*P*	*I*^*2*^					
LOS	3	191/230	10.02	0.007	80%	Random	15.28 [1.11, 29.46] ^a^	2.11	0.03*	0.329
Prior hospitalization (within the previous 6 months)	4	230/172	5.67	0.13	47%	Fixed	1.91 [1.23, 2.97]	2.89	0.004*	0.480
Admission to ICU	10	684/874	31.80	0.0002	72%	Random	3.20 [1.97, 5.18]	4.72	<0.00001*	0.796
Length of ICU stay	3	259/196	4.84	0.09	59%	Random	−1.78 [−9.25, 5.68] ^a^	0.47	0.64	0.909
APACHE II score on ICU admission	5	253/157	10.95	0.03	63%	Random	0.91 [−1.28, 3.10] ^a^	0.82	0.41	0.692
Hypertension	3	148/200	0.08	0.96	0%	Fixed	0.97 [0.61, 1.55]	0.12	0.91	0.271
Diabetes	13	757/800	10.80	0.55	0%	Fixed	1.12 [0.88, 1.43]	0.94	0.35	0.874
Respiratory disease	3	149/118	0.77	0.68	0%	Fixed	1.34 [0.71, 2.55]	0.91	0.37	0.294
Heart disorders	5	305/290	3.79	0.44	0%	Fixed	1.25 [0.87, 1.78]	1.20	0.23	0.594
Acute renal failure	3	261/198	0.95	0.62	0%	Fixed	1.12 [0.68, 1.85]	0.46	0.65	0.156
Chronic renal failure	6	460/494	7.24	0.20	31%	Fixed	1.25 [0.89, 1.73]	1.30	0.19	0.580
Renal dysfunction	3	213/279	2.26	0.32	11%	Fixed	2.17 [1.32, 3.56]	3.07	0.002*	0.072
Liver disease	5	313/243	2.17	0.70	0%	Fixed	1.40 [0.88, 2.23]	1.43	0.15	0.665
Neurological disorders	5	289/235	1.51	0.83	0%	Fixed	1.52 [1.04, 2.24]	2.15	0.03*	0.081
Hematological disorders	3	177/122	0.78	0.68	0%	Fixed	2.83 [0.82, 9.72]	1.65	0.10	0.772
Malignancy	5	343/374	5.50	0.24	27%	Fixed	0.84 [0.55, 1.28]	0.82	0.41	0.306
Trauma	3	168/219	0.82	0.66	0%	Fixed	0.58 [0.30, 1.12]	1.63	0.10	0.324
Immunosuppression	3	135/124	2.25	0.32	11%	Fixed	1.49 [0.71, 3.13]	1.04	0.30	0.106
Steroid therapy	3	174/174	1.09	0.58	0%	Fixed	1.44 [0.85, 2.44]	1.34	0.18	0.108
Chemotherapy	3	148/187	0.16	0.92	0%	Fixed	1.03 [0.47, 2.26]	0.07	0.95	0.169
Prior surgery	11	616/628	22.47	0.01	55%	Random	1.31 [0.88, 1.94]	1.33	0.18	0.723
Tracheostomy	6	385/468	18.30	0.003	73%	Random	2.11 [1.03, 4.32]	2.05	0.04*	0.769
Mechanical ventilation	12	764/947	41.95	<0.0001	74%	Random	2.70 [1.68, 4.33]	4.12	<0.0001*	0.901
CVC	9	642/706	30.00	0.0002	73%	Random	2.62 [1.44, 4.76]	3.16	0.002*	0.871
Urinary catheter	10	532/606	22.30	0.008	60%	Random	1.99 [1.28, 3.09]	3.04	0.002*	0.626
Nasogastric tube	6	250/246	17.20	0.004	71%	Random	2.62 [1.20, 5.68]	2.43	0.02*	0.623
Dialysis	7	378/527	3.01	0.81	0%	Fixed	3.56 [2.39, 5.31]	6.25	<0.00001*	0.592
Parenteral nutrition	4	231/178	6.64	0.08	55%	Random	1.59 [0.72, 3.49]	1.15	0.25	0.448
Enteral feeding	3	178/130	2.02	0.36	1%	Fixed	1.35 [0.78, 2.35]	1.08	0.28	0.843
Prior antibiotic use	6	352/507	20.64	0.0009	76%	Random	6.07 [2.03, 18.18]	3.22	0.001*	0.133
Penicillin	3	185/282	7.07	0.03	72%	Random	2.18 [0.75, 6.35]	1.42	0.15	0.408
Cephalosporins	7	468/513	31.51	<0.0001	81%	Random	1.45 [0.70, 2.99]	1.00	0.32	0.148
Second-generation cephalosporins	3	149/135	0.13	0.94	0%	Fixed	1.62 [0.75, 3.47]	1.23	0.22	0.357
Third-generation cephalosporins	3	112/157	4.61	0.10	57%	Random	2.05 [0.83, 5.06]	1.56	0.12	0.756
Carbapenems	12	658/774	25.57	0.008	57%	Random	4.16 [2.75, 6.29]	6.76	<0.00001*	0.954
β-lactam+β-lactamase inhibitor	5	262/273	13.21	0.01	70%	Random	2.06 [1.01, 4.20]	2.00	0.05	0.276
Aminoglycosides	12	669/765	10.47	0.49	0%	Fixed	1.85 [1.32, 2.60]	3.54	0.0004*	0.770
Quinolones	8	420/531	20.85	0.004	66%	Random	2.11 [1.15, 3.87]	2.42	0.02*	0.324
Fluoroquinolones	4	249/234	0.57	0.90	0%	Fixed	2.03 [1.28, 3.24]	2.98	0.003*	0.184
Glycopeptides	4	191/230	0.69	0.88	0%	Fixed	3.70 [2.31, 5.94]	5.43	<0.00001*	0.677
Vancomycin	3	195/292	3.64	0.16	45%	Fixed	2.82 [1.86, 4.28]	4.87	<0.00001*	0.930
Macrolides	4	254/404	10.12	0.02	70%	Random	2.46 [0.44, 13.87]	1.02	0.31	0.571
Metronidazole	4	201/240	0.52	0.92	0%	Fixed	0.85 [0.50, 1.43]	0.62	0.54	0.491

*Abbreviations*: *CRKP* Carbapenem-resistant *Klebsiella pneumoniae*, *CSKP* Carbapenem-susceptible *Klebsiella pneumoniae*, *OR* Odds ratio, *MD* Mean difference, *CI* Confidence interval, *LOS* Length of hospital stay, *ICU* Intensive care unit, *APACHE* Acute Physiology and Chronic Health Evaluation, *CVC* Central venous catheter

^a^Mean difference

* Statistically significant differences between groups (α = 0.05)

#### Risk factors for CRKP infection compared with absence of CRKP infection (comparison 2)

Table 4 shows the risk factors for CRKP infection for this comparison, as well as the heterogeneity in the meta-analysis. All 20 risk factors were dichotomous variables, and the following eight were statistically significant: admission to ICU, tracheostomy, mechanical ventilation, CVC use, urinary catheter use, prior antibiotic use, and specific use of carbapenems or aminoglycosides.

**Table 4:** Meta-analysis of risk factors for CRKP infection in the type 2 comparison

	Number of included studies	Sample size (CRKP infection/Without CRKP infection)	Heterogeneity			Effects model	OR [95% *CI*]	*Z*	*P*	Egger’s test *P* >|t|
			χ^*2*^	*P*	*I*^*2*^					
Admission to ICU	4	576/1572	41.44	<0.00001	93%	Random	4.44 [1.32, 14.95]	2.40	0.02*	0.313
Diabetes	6	523/1718	6.59	0.25	24%	Fixed	1.36 [0.99, 1.86]	1.92	0.05	0.199
Hypertension	3	94/860	3.83	0.15	48%	Fixed	1.06 [0.65, 1.72]	0.23	0.82	0.127
HBV	3	39/497	1.41	0.49	0%	Fixed	0.79 [0.31, 2.02]	0.50	0.62	0.116
HCV	4	86/613	3.88	0.27	23%	Fixed	1.41 [0.85, 2.34]	1.33	0.19	0.083
HCC	4	86/613	7.78	0.05	61%	Random	1.14 [0.43, 3.02]	0.26	0.80	0.488
Alcoholic liver disease	3	78/311	0.23	0.89	0%	Fixed	1.13 [0.65, 1.97]	0.44	0.66	0.555
Retransplantation	3	54/571	7.39	0.02	73%	Random	3.70 [0.74, 18.58]	1.59	0.11	0.590
Tracheostomy	3	161/245	0.17	0.92	0%	Fixed	8.48 [4.43, 16.22]	6.46	<0.00001*	0.375
Mechanical ventilation	5	693/1539	67.27	<0.00001	94%	Random	4.78 [1.78, 12.82]	3.10	0.002*	0.652
CVC	4	632/1473	34.74	<0.00001	91%	Random	3.85 [1.56, 9.52]	2.92	0.004*	0.996
Urinary catheter	5	693/1539	108.70	<0.00001	96%	Random	0.27 [0.02, 0.51]	2.13	0.03*	0.748
Dialysis	3	164/195	0.48	0.79	0%	Fixed	1.54 [0.86, 2.75]	1.47	0.14	0.158
Parenteral nutrition	3	262/733	7.89	0.02	75%	Random	1.73 [0.80, 3.74]	1.39	0.16	0.966
Prior antibiotic use	4	253/1051	3.95	0.27	24%	Fixed	1.61 [1.05, 2.48]	2.19	0.03*	0.265
Carbapenems	5	627/1635	22.29	0.0002	82%	Random	3.84 [2.02, 7.28]	4.12	<0.0001*	0.222
β-lactam+β-lactamase inhibitor	3	537/1373	58.55	<0.00001	97%	Random	1.89 [0.48, 7.48]	0.91	0.37	0.538
Aminoglycosides	4	585/1551	3.50	0.32	14%	Fixed	1.80 [1.28, 2.55]	3.34	0.0008*	0.415
Fluoroquinolones	3	533/1529	14.90	0.0006	87%	Random	1.71 [0.77, 3.77]	1.33	0.18	0.904
Glycopeptides	3	215/811	1.66	0.44	0%	Fixed	1.44 [0.96, 2.14]	1.78	0.07	0.812

*Abbreviations*: *CRKP* Carbapenem-resistant *Klebsiella pneumoniae*, *OR* Odds ratio, *CI* Confidence interval, *ICU* Intensive care unit, *HBV* Hepatitis B virus, *HCV* Hepatitis C virus, *HCC* Hepatocellular carcinoma, *CVC* Central venous catheter

* Statistically significant differences between groups (α = 0.05)

### Publication bias

Egger’s test showed no obvious asymmetry in the risk factors, suggesting low risk of publication bias (Tables 3 and 4).

### Sensitivity analyses

The sensitivity analysis was performed by repeating the meta-analysis after omitting each study one by one and examining whether the results changed substantially. For most risk factors, no single study seemed to substantially alter the results. We noted two exceptions: in comparison 1, omitting the study by Mouloudi et al. from 2010 [30] made the factor “β-lactam + β-lactamase inhibitor” significant (OR 2.42, 95% CI 1.08 to 5.44); in comparison 2, removing the study by Mouloudi et al. in 2014 [37] made the factor “diabetes” significant (OR 1.39, 95% CI 1.01 to 1.90).

## Discussion

CRKP is one of the most serious life-threating nosocomial pathogens worldwide, and CRKP infections are highly prevalent in most of the countries where the studies included in our review were performed (such as Italy, China, Greece, USA, Turkey and Israel). The proportion *K. pneumoniae* infections involving meropenem resistance in China increased from 14.1% in 2013 to 28.6% in 2018, with four provinces showing CRKP proportions > 10% in 2013 (the highest was Zhejiang province with 37.40%) and 13 in 2017 (the highest was Henan province with 53.01%) [49]. The proportion of *K. pneumoniae* infections involving meropenem resistance has grown steeply in the USA from 0.6% in 2004 to 10.8% in 2007 [50]. The most severely affected European countries are Greece and Italy, where 64.7 and 29.7% of *K. pneumoniae* infections in 2017 showed carbapenem resistance [3]. The proportion of CRKP infections in Turkey increased from 3.2% in 2010 to 66.9% in 2014 [39]. Israel faced a nationwide CRKP outbreak in 2006 that, by mid-2007, had infected 1275 patients in 27 hospitals [51]. The identification of risk factors of CRKP is the first step to discover high-risk patients and high-risk wards in order to channel limited resources most effectively into prevention and treatment.

Unfortunately, although many studies have investigated risk factors for CRKP infection, they have come to diverging, often conflicting, conclusions. For example, some studies have reported that exposure to carbapenems increased the risk of CRKP infection [17–22, 27, 29, 31, 33], but others did not find the same effect [24, 30]. These discrepancies may reflect differences in sample size and overall lack of statistical power, which prompted us to perform a systematic review in order to assess the associations as reliably and comprehensively as possible.

We based our review on the idea that the choice of the control group for risk assessment can provide different results, as suggested in several previous studies [9–12]. We meta-analyzed 32 studies in nine countries involving several thousands of patients. Consistent with our initial idea, the profiles of risk factors differed between comparisons 1 and 2, with immediate implications for clinical practice. Comparison 1 assessed risk factors for carbapenem-resistant infections, which are relevant for the situation when the patient is known to be infected with *K. pneumoniae* but tests of antibiotic susceptibility are pending. In this case, the clinician estimates the probability of resistance to carbapenem based on risk factors, adopting an empirical approach that prioritizes interventions to prevent transmission of carbapenem resistance at this early stage. In this type of comparison, our analysis identified the following risk factors: prior hospitalization (within the previous 6 months), longer length of stay, admission to the ICU, concomitant diseases (renal dysfunction, neurological disorders), certain invasive procedures (tracheostomy, mechanical ventilation, CVC, urinary catheter, nasogastric tube and dialysis), prior use of any antibiotic, and specific exposure to vancomycin or other five classes of antimicrobial agents (carbapenems, aminoglycosides, quinolones, fluoroquinolones, glycopeptides). These risk factors are more likely to be present in patients with more severe illness and greater susceptibility to infection, and who are therefore exposed to greater antibiotic selection pressure, which may ultimately increase the likelihood of infection with multidrug-resistant pathogens [20].

Comparison 2 is more relevant for the situation when hospitals need to identify patients at increased risk of suffering *K. pneumoniae* infection in general and CRKP in particular. The impact of risk factors on CRKP infection reflects an integrated effect of *K. pneumoniae* characteristics and carbapenem resistance. This may allow clinicians and hospital epidemiologists to take timely action to prevent CRKP transmission, even when no pathogen is detected in patient specimens, which may be due to their use of medications. In this type of comparison, our analysis identified the following risk factors: admission to ICU, certain invasive procedures (tracheostomy, mechanical ventilation, CVC, urinary catheter), prior use of any antibiotic, and exposure to carbapenems or aminoglycosides. Importantly, these risk factors were also statistically significant in comparison 1, which means that they are probably true risk factors for acquiring CRKP infection among hospitalized patients.

In contrast, dialysis and exposure to fluoroquinolones or glycopeptides were risk factors only for the first comparison. These factors may therefore increase primarily the risk of carbapenem resistance in *K. pneumoniae.* Indeed, fluoroquinolone exposure can generate resistance not only to fluoroquinolones but also to carbapenems, as fluoroquinolones lead to upregulation of the multidrug efflux pump MexEF-OprN and downregulation of the porin OprD, which is involved in carbapenem resistance [51, 52]. In addition, a quinolone resistance gene that causes low-level fluoroquinolone resistance is located on *K. pneumoniae* plasmids carrying carbapenemase genes [52]. Long-term administration of the glycopeptide vancomycin may disrupt the balance of microflora in the body, promoting the propagation of Gram-negative bacteria and increasing the rate of mutation and spread of carbapenemases, which may augment the risk of CRKP [18]. These considerations imply that restricting the use of fluoroquinolones and glycopeptides, whenever possible, may decrease the transmission of carbapenem resistance.

Our sensitivity analysis confirmed that meta-analysis results were robust, with the possible exceptions of exposure to β-lactam + β-lactamase inhibitor (comparison 1) and diabetes (comparision 2). The status of these variables as risk factors changed depending on the inclusion of two small studies [30, 37]. The heterogeneity surrounding these variables suggests the need for further studies to confirm their relationship with risk of CRKP infection.

Compared to a previous meta-analysis with a similar goal [8], the present work included 12 additional studies involving 2981 patients published after September 2016. In addition, we excluded studies comparing patients infected with CPKP with controls without CPKP infection, and our results for separate two comparisons contrast with a previous meta-analysis that aggregated both types of comparison. Consistent with our initial hypothesis, we identified several differences in the risk factors that were significant in each comparison, and we were able to derive a set of likely true risk factors of CRKP infection as those factors significant in both comparisons. The previous work identified the following significant risk factors: exposure to glycopeptides, parenteral nutrition, length of ICU stay and steroid therapy [8]. In our analysis, however, exposure to glycopeptides was significant only in comparison 1, while length of ICU stay and steroid therapy were not significant in comparison 1, and parenteral nutrition was not significant in either type of comparison, suggesting that these four factors may not be considered true risk factors. Furthermore, we found urinary catheter use to be a significant risk factor in both types of comparison, contrary to the previous meta-analysis.

Like the previously published meta-analysis on risk factors of CRKP infection [8], our exclusion criteria did not include that the source or base population of both case and control groups were identified with CRKP colonization based on rectal culture. With the exception of two studies [35, 36], the studies included in our meta-analysis did not perform rectal screening for CRKP, and thus potential CRKP rectal colonization was not identified. In these cases, it was difficult to judge whether the risk factors associated with the process of CRKP colonization developing into infection or acquiring CRKP and having it cause infection. Moreover, the relative timing of CRKP colonization and onset of risk factors is often difficult to determine [36]. Further studies are needed in which risk factors associated with CRKP colonization developing into infection, which would then allow meta-analysis to identify the risk factors for CRKP infection among patients with CRKP colonization.

The findings of our meta-analysis should be interpreted with caution given that some potential risk factors were analyzed based on data from a small number of studies. Indeed, data for some factors showed significant heterogeneity across studies, especially in comparison 2, probably because control patients included those without any infection as well as those infected with nosocomial pathogens other than CRKP. Most studies in our review were retrospective and all were observational, increasing the risk of patient selection bias, outcome reporting bias and confounding. Nevertheless, all studies received NOS scores indicating high quality, and no obvious publication bias was observed for any of the factors. Factors affecting risk of CRKP infection should be further examined in large, well-controlled prospective studies.

## Conclusions

This meta-analysis identified 18 factors that increase the risk of carbapenem resistance in *K. pneumoniae* infection and eight factors which were associated with both *K. pneumoniae* infections in general and CRKP in particular. The eight shared factors are probably ‘true’ risk factors for CRKP infection. These findings may help clinicians and hospital epidemiologists estimate the likelihood of CRKP infection in different situations, and thereby initiate timely, targeted treatment and prevention measures.

## Data Availability

The datasets supporting the conclusions of this article are included with in the article (Tables 1, 2, 3 and 4).

## Abbreviations

APACHE: Acute Physiology and Chronic Health Evaluation
*CI*: Confidence interval
CPKP: Carbapenemase-producing *Klebsiella pneumoniae*
CRKP: Carbapenem-resistant *Klebsiella pneumoniae*
CSKP: Carbapenem-susceptible *Klebsiella pneumoniae*
CVC: Central venous catheter
HBV: Hepatitis B virus
HCC: Hepatocellular carcinoma
HCV: Hepatitis C virus
ICU: Intensive care unit
LOS: Length of hospital stay
MD: Mean difference
NA: Not available
NOS: Newcastle-Ottawa Scale
OR: Odds ratio
PRISMA: Preferred Reporting Items for Systematic Reviews and Meta-Analyses
SD: Standard deviation
